# Paternal smoking and maternal secondhand smoke exposure and the effects on the offspring: results from the EHF (Environmental Health Fund) birth cohort

**DOI:** 10.1186/s13584-025-00706-3

**Published:** 2025-07-08

**Authors:** Maya Berlin, Elkana Kohn, Rimona Keidar, Ayelet Livne, Ronella Marom, Amit Ovental, Dror Mandel, Ronit Lubetzky, Moshe Betser, Miki Moskovich, Ariela Hazan, Ludmila Groisman, Efrat Rorman, Matitiahu Berkovitch, Ilan Matok, Laura J. Rosen

**Affiliations:** 1 Clinical Pharmacology and Toxicology Unit, Shamir Medical Center (Assaf Harofeh), Zerifin, Affiliated to Faculty of Medical and Health Sciences, Tel-Aviv University, Tel Aviv, 7033001 Israel; 2 Division of Clinical Pharmacy, Institute for Drug Research, School of Pharmacy, Faculty of Medicine, Hebrew University of Jerusalem, Jerusalem, Israel; 3 Department of Neonatology, Shamir Medical Center (Assaf Harofeh), Zerifin, Affiliated to Faculty of Medical and Health Sciences, Tel-Aviv University, Tel Aviv, Israel; 4 Departments of Neonatology and Pediatrics, Dana Dwek Children’s Hospital, Tel Aviv Medical Center, Affiliated to Faculty of Medical and Health Sciences, Tel-Aviv University, Tel Aviv, Israel; 5 Division of Obstetrics and Gynecology, Shamir Medical Center (Assaf Harofeh), Zerifin, Affiliated to Faculty of Medical and Health Sciences, Tel-Aviv University, Tel Aviv, Israel; 6 National Public Health Laboratory, Ministry of Health, Tel Aviv-Yafo, Israel; 7 Department of Health Promotion, School of Public Health, Faculty of Medical and Health Sciences, Tel Aviv University, Tel Aviv, Israel

**Keywords:** Secondhand smoke, Environmental tobacco smoke, Urinary cotinine, Human biomonitoring, Prenatal exposure

## Abstract

**Background:**

Exposure during pregnancy to secondhand smoke (SHS) is associated with a wide range of adverse fetal development effects and perinatal outcomes. This study aimed to measure SHS exposure in pregnant women using urinary cotinine measurements and to evaluate the association between paternal smoking and maternal urine cotinine and birth outcomes in a birth cohort of Israeli mothers and children.

**Methods:**

We measured cotinine in urine samples taken at birth from 96 non-smoking mothers participating in the EHF (Environmental Health Fund)-Assaf-Harofeh-Ichilov cohort. Half of the partners were smokers, based on the self-reported data. Logistic regression models were used to predict maternal urinary cotinine levels, and potential confounders were included in the model. We analyzed the association between maternal urinary cotinine levels and birth weight.

**Results:**

In our study of 96 nonsmoking pregnant women, 94% of women with a smoking partner and nearly 60% of those with a non-smoking partner were exposed to SHS and had urine cotinine levels above the Limits of Quantification (LOQ). Paternal smoking was a significant predictor for maternal urinary cotinine levels above the LOQ (aOR 7.83 95%CI [2.01–30.57], p-value = 0.003). There were no statistically significant differences between the birth outcomes (e.g. gestational age, birth weight, birth length, head circumference, and the delivery method) tested in both groups– self-reported smoking status and exposed vs. non-exposed newborns. Maternal urinary cotinine levels were inversely associated with newborns’ birth weight (beta estimate − 281.39, p-value = 0.048). When stratified by sex, in male newborns, maternal urinary cotinine was inversely associated with newborns’ birth weight (beta estimate 470.22, p-value = 0.014), but not in females.

**Conclusions:**

Our findings support the fact that partners are one of the primary sources of maternal secondhand smoke and may play an essential role in creating and maintaining smoke-free environments.

**Trial registration:**

ClinicalTrials.gov, ID: NCT03084445. Registered 06 February 2014, https://clinicaltrials.gov/study/NCT03084445.

**Supplementary Information:**

The online version contains supplementary material available at 10.1186/s13584-025-00706-3.

## Introduction

The WHO (World Health Organization) has classified the tobacco epidemic as one of the biggest public health threats worldwide [1]. Cigarette smoking during pregnancy is associated with adverse birth outcomes such as spontaneous abortion, low birth weight, and preterm birth. These adverse birth outcomes negatively impact children’s health and development [2].

Secondhand smoke (SHS), also known as environmental tobacco smoke (ETS), is a mixture of the smoke from the burning tobacco product and the smoke exhaled by a smoker [3]. According to the U.S. Centers for Disease Control and Prevention, there is no safe level of SHS exposure. Even brief exposures can be harmful [4]. Newborn infants and fetuses are especially susceptible to the harms of SHS exposure [5].

Exposure during pregnancy to SHS is associated with a wide range of adverse fetal development effects and perinatal outcomes, as well as higher infant mortality. Evidence suggests maternal smoking or secondhand smoke exposure during pregnancy is linked to birth defects, stillbirths, preterm births, and infant deaths [6]. Exposure to SHS during pregnancy is associated with up to 23% increased risk of stillbirth and up to 13% increased risk of congenital malformation [6, 7]. Paternal smoking around conception or during pregnancy is associated with an increased risk of acute lymphoblastic leukemia in children [8].

Previous studies indicate widespread exposure to SHS in non-smoking adults and children in Israel. According to the National Survey in 2015–2016, over 60% of adult non-smokers and children in Israel were exposed to ETS, based on urinary cotinine measurements [9, 10].

The “Jerusalem Environment Mother and Child” Cohort Study previously reported that about 35% of pregnant women and about 30% of newborns were exposed to SHS exposure. Perinatal SHS exposure was associated with reduced birth weight and head circumference in newborns [11].

Cotinine, the primary metabolite of nicotine, is considered a biomarker of exposure to SHS, and cotinine assays provide an objective and more reliable quantitative measure than smoking history or counting the number of daily cigarettes [12]. Urinary cotinine is the biomarker of choice for quantifying SHS exposure [13]. Cotinine has a longer biological half-life (16–18 h) relative to nicotine, and its levels remain constant during the day. Urinary cotinine reflects exposure to SHS in the past few days [14].

This study aimed to measure SHS exposure in pregnant women using urinary cotinine measurements and to evaluate the association between paternal smoking and maternal urine cotinine and birth outcomes in a birth cohort of Israeli mothers and children.

## Methods

### Study population

We used data from a birth cohort recruited at Shamir Medical Center (Assaf Harofeh) and “Dana Dwek” Children’s Hospital, Tel Aviv Medical Center. Complete details of this birth cohort have been presented elsewhere [15, 16].

From January 2013 through April 2015, 263 mother-father-newborn triads were recruited. The women recruited for the study were asked to participate during their time in the delivery room. Data on social and demographic characteristics and lifestyle variables from both the father and mother were obtained through a detailed questionnaire, filled in separately. Data on smoking habits, occupation, residential history, diet, hobbies, and detailed health history were also collected. Maternal blood and urine samples were collected in the delivery room.

Birth weight, length, and head circumference were measured three times using standard research procedures; values were averaged.

All participants signed informed consent. The study was performed according to the Declaration of Helsinki, and the Institutional Helsinki Committee approved the protocol [17].

### Data extraction

The database included 263 mother-newborn dyads. Cases of women with twin pregnancies (*N* = 2), premature delivery (< 37 weeks) (*N* = 8), and incomplete details from questionnaires (*N* = 3) were not selected for analysis, leaving 250 cases. Due to budget limitations, we randomly selected 99 nonsmoking women out of 250 women. According to the questionnaire’s self-reported smoking status, half of the partners were smoking.

Two out of 99 cases were excluded due to misclassification of maternal smoking status, and 1 case was excluded due to an incomplete paternal questionnaire. See Supplementary Fig. 1 for a Flowchart of the study population.

We retrieved data on 96 dyads for which urinary cotinine measurements were obtained. Data on maternal demographics, exposures, lifestyle, and labor were extracted from the questionnaires, as well as newborn anthropometrics and measurements.

### Sample analysis

Urinary cotinine and creatinine were measured in 99 pregnant women. Maternal samples were collected at the time of delivery at Shamir Medical Center (Assaf Harofeh) in polypropylene tubes and stored at − 80 °C until analysis between January 2013 through April 2015.

Cotinine analysis was performed using a gas chromatography-mass spectrometry procedure validated and published by the German Research Foundation (DFG) working group “Analyses in biological materials” [18]. Cotinine was extracted by dichloromethane from urine samples and spiked with isotope-labeled analog used as an internal standard. The final extract was injected into Agilent 7000GC/MS Triple Quad Instrument and analyzed in MRM Mode.

The method followed standard quality assurance and quality control procedures. Urinary cotinine was quantified using an internal standard calibration procedure and certified analytical standards. Quality control was performed by analyzing aliquots of control material (every ten samples) purchased from G-EQUAS, and accuracy was validated by the successful annual participation in the international proficiency test (G-EQUAS) for all parameters. Limits of quantification (LOQ) for cotinine in urine was 0.5 ng/mL. Urine Cotinine concentrations below LOQ were assigned half of the LOQ (= 0.25 ng/mL).

Urine creatinine levels were measured on COBAS 8000 autoanalyzer (ROCHE Diagnostic, IN, USA) according to the manufacturer’s instructions at the Biochemistry Laboratory at the Shamir Medical Center. It was used to standardize the cotinine concentration detected in the urine samples by a simple adjustment and a covariate in statistical models, as suggested by O’Brien et al. [19, 20].

Urinary cotinine concentrations were provided in units of ng/mL; creatinine-adjusted concentrations were provided as g creatinine/L urine.

### Statistical analysis

In the statistical analysis, we treated all unadjusted (ng/mL) and creatinine-adjusted (mcg/g) values as continuous variables. In addition, we conducted analyses with cotinine as a dichotomous variable– below or above the LOQ. We classified women as exposed or non-exposed to secondhand smoke based on urinary cotinine below or above the LOQ. The percent of women with urinary cotinine above the LOQ was calculated, and the geometric mean and median of cotinine were calculated as adjusted and unadjusted to maternal urinary creatinine.

Other continuous variables are presented as mean and standard deviation (± SD) or median and interquartile range (IQR). The distribution of continuous variables was assessed using visual inspection methods, including histograms and quantile–quantile (Q–Q) plots, to evaluate normality. Independent t-tests were used only for variables that were approximately normally distributed, including maternal age, maternal total education years, paternal age, and paternal total education years. For all other continuous variables that did not meet the normality assumption (e.g., birth weight, birth length, gestational age, and head circumference– Supplementary Table 1), we used the Mann–Whitney U test.

Categorical variables were compared using the Chi-square test, or Fisher’s exact test, as appropriate.

Spearman correlation coefficients were calculated to assess the correlation between continuous variables of the mother and the newborn and maternal urinary cotinine levels.

Maternal and paternal demographic characteristics, anthropometric measurements, and newborns’ characteristics were presented. In addition, we collected information on self-reported paternal smoking status (reported by the father) (smoking vs. non-smoking partner).

We used multivariable models to assess exposure as (1) a continuous variable, based on urinary cotinine level, and (2) a dichotomous variable, based on the LOQ.

1. Multivariate linear regression models were performed to analyze the associations between maternal urinary cotinine level (as a continuous variable) with birth weight. All models were adjusted for maternal urinary creatinine, paternal smoking status, maternal age, gestational age, maternal pre-pregnancy BMI, and newborn sex. We assessed the assumptions of linear regression by examining model residuals. Residuals were visually inspected using histograms and Q–Q plots and were found to be approximately normally distributed, without evidence of heteroscedasticity. These findings support the use of linear regression in our cohort. Because previous studies suggest differences in possible effects of prenatal exposure to SHS between boys and girls, such as anogenital distance [21] or the risk of obesity in early adolescence [22], a further analysis was conducted which was stratified by the newborn sex.
2. Logistic regression models were performed to predict the maternal urinary cotinine levels (as dichotomous variables, above and below LOQ). The potential confounders included in the model were: paternal and maternal age, paternal smoking status, paternal education status academic vs. non-academic, and monthly household income (Net). The paternal education status was classified as academic if the father reported completing a bachelor’s degree or higher. Paternal education was classified as non-academic for any education below a completed bachelor’s degree (including high school or professional courses). We present an adjusted Odds Ratio (aOR) with confidence intervals (CI) for each potential confounder.

All statistical tests were two-sided; p. < 0.05 was considered statistically significant. SPSS software (IBMS SPSS Statistics for Windows, Version 27, IBM Corp, Armonk, New York, USA) was used for all statistical analyses.

## Results

### Subjects’ characteristics

Ninety-six mother-father-newborn triads were included in the final analysis. Three cases were excluded from the final analysis due to misclassification of maternal smoking status (*N* = 2) and incomplete paternal questionnaire (*N* = 1). Maternal and paternal demographic characteristics of self-reported paternal smoking status (smoking partner vs. non-smoking partner and exposure status (urinary cotinine above LOQ = 0.5 ng/mL) vs. non-exposed (urinary cotinine below LOQ = 0.5 ng/mL) are presented in Table 1. There were no statistically significant differences between the parameters tested in both groups– paternal self-reported smoking status, and exposed vs. non-exposed women. Maternal total education years were slightly higher in the non-smoking partner group (16 vs. 15.9, p-value 0.038). In the non-exposed group, 19 partners (86.4%) reported being non-smokers, whereas, in the exposed group, 26 partners (35.1%) reported being non-smokers, with a p-value < 0.001.

**Table 1:** Maternal and paternal demographic characteristics in the EHF-Assaf-Harofeh-Ichilov birth cohort, Israel, 2013–2015

	Self-reported smoking status per questionnaire			Maternal Urinary Cotinine Below or Above LOQ (0.5 ng/mL)		
Maternal characteristics	Nonsmoking father	Smoking father	*P* value	Non-exposed	Exposed	*P* value
**Number**	**45**	**51**		**22**	**74**	
Maternal age at delivery (years) Mean ± SD	32.9 ± 4.79	31.44 ± 4.79	0.138	33.6 ± 5.3	31.7 ± 4.6	0.107
Planned pregnancy (yes) (N, %)	34 (75.6%)	42 (82.4%)	0.413	16 (72.7%)	60 (81.1%)	0.387
**Parity**						
Primiparous (yes) (N, %)	12 (26.7%)	10 (19.6%)	0.412	5 (22.7%)	18 (24.7%)	0.853
**Education/Social/Habits**						
Maternal total education years (mean ± SD)	16.0 (± 2.65)	14.9 (± 2.37)	**0.038**	16.05 (± 2.87)	15.24 (± 2.44)	0.197
Non-academic (N, %)	13 (28.9%)	22 (44%)	0.127	6 (27.3%)	29 (39.7%)	0.288
Academic (N, %)	32 (71.1%)	28 (56%)		16 (72.7%)	44 (60.3%)	
Employed (yes) (N, %)	39 (86.7%)	44 (86.3%)	0.955	19 (86.4%)	64 (86.5%)	1
Mother Former smoker (N, %)	5 (11%)	12 (23.5%)	0.19	2 (9.1%)	15 (20.3%)	0.344
Has Ever Consumed Alcohol During Pregnancy (yes) (N, %)	1 (2.2%)	5 (9.8%)	0.209	0	6 (8.1%)	0.331
Multi-unit building living (yes) (N, %)	34 (75.6%)	39 (76.5%)	0.917	18 (81.8%)	55 (74.3%)	0.47
**Monthly Household Income (Net)**						
Less than 8500–9000 NIS (yes) (N, %)	13 (31.7%)	20 (41.7%)	0.332	6 (30%)	27 (39.1%)	0.457
Above or equal 8500–9000 (yes) (N, %)	28 (68.3%)	28 (58.3%)		14 (70%)	42 (60.9%)	
**Country of origin**						
Both parents Israeli born (N, %)	38 (84.4%)	37 (72.5%)	0.159	19 (86.4%)	56 (75.7%)	0.385
**Paternal characteristics**						
Paternal age at delivery (years) Mean ± SD	35.67 ± 5.52	34.0 ± 4.59	0.146	36.45 ± 5.03	34.29 ± 5.62	0.109
**Education/Social/Habits**						
Paternal total education years (mean ± SD)	15.07 ± 2.92	14.31 ± 2.78	0.199	15.09 ± 2.79	14.54 ± 2.88	0.43
Non-academic (N, %)	19 (43.2%)	29 (56.9%)	0.184	7 (33.3%)	41 (55.4%)	0.074
Academic (N, %)	25 (56.8%)	22 (43.1%)		14 (66.7%)	33 (44.6%)	
**Paternal Smoking Patterns**						
Non-smoker	45 (100%)	0	**< 0.001**	19 (86.4%)	26 (35.1%)	**< 0.001**
Cigarettes and/or Nargila	0	51 (100%)		3 (13.6%)	48 (64.9%)	

**Bold** - groups with a statistically significant difference

IQR - interquartile range; SD - Standard Deviation, LOQ - Limits of quantification

Anthropometric measurements and characteristics of the newborns were presented for self-reported paternal smoking status (smoking vs. non-smoking partner) and for exposed (urinary cotinine above LOQ = 0.5 ng/mL) vs. non-exposed (urinary cotinine below LOQ = 0.5 ng/mL) in Supplementary Table 1. There were no statistically significant differences between the parameters tested in both groups– self-reported smoking status and exposed vs. non-exposed newborns. The mean gestational age for all groups was 39.1-39.56 weeks (SD: 1.23–1.28). The mean birth weight for all groups was 3190–3254 g (SD: 410–510).

### SHS exposure measurement in pregnant women using urinary cotinine

Maternal urinary cotinine levels were significantly higher in women with smoking partners. The geometric mean of urinary cotinine (ng/mL) in women with non-smoking partners was 0.57 (± 0.50), compared to 1.07 (± 1.17) in women with smoking partners (p-value = 0.005) (Table 2). The same trend was observed for urine cotinine adjusted to creatinine (mcg/g creatinine) - geometric mean (± SD) for women with a non-smoking partner was 1.16 (± 2.1), whereas, in women with a smoking partner, the geometric mean was 2.09 (± 2.49), p-value < 0.001.

**Table 2:** Maternal urinary cotinine levels in the EHF-Assaf-Harofeh-Ichilov birth cohort, Israel, 2013–2015

		Self-reported smoking status per questionnaire		
Maternal measurements		Nonsmoking partner	Smoking partner	*P* value
**N**	**96**	**45**	**51**	
Urine cotinine (ng/mL) Geometric mean ± SD Median (IQR)	0.79 ± 0.96 1.62 (0.77–3.24)	0.57 ± 0.50 0.68 (0.25–1.16)	1.07 ± 1.17 1.13 (0.75–1.42)	**0.005**
Urine cotinine adjusted to creatinine (mcg/g creatinine) Geometric mean ± SD Median (IQR)	1.58 ± 2.35 0.86 (0.623–1.29)	1.16 ± 2.1 1.06 (0.46–2.97)	2.09 ± 2.49 2.16 (1.27–3.47)	**< 0.001**
Above LOQ (N, %)	74 77.1%	26 57.8%	48 94.1%	**< 0.001**

SD - Standard Deviation, LOQ - Limits of quantification, IQR - interquartile range.

**Bold** - groups with a statistically significant difference

Almost all women with a smoking partner had urinary cotinine levels above the LOQ = 94.1% (*N* = 48). By contrast, 57.8% (*N* = 26) of women with a non-smoking partner had urinary cotinine levels above the LOQ (p-value < 0.001).

Logistic regression analysis examined factors affecting maternal exposure as assessed by urinary cotinine (exposed vs. unexposed, based on LOQ) (Table 3). Maternal urinary cotinine (above the LOQ) was significantly associated with paternal smoking (aOR 7.83 95%CI [2.01–30.57], p-value = 0.003). Paternal education was a predictor of urinary cotinine levels below the LOQ in the multivariable model: while controlling for paternal smoking status, women with partners with academic education had urinary cotinine levels which were more often below the LOQ than women with partners without academic education: aOR 0.27 95%CI [0.072–1.02], p-value = 0.053.

**Table 3:** Logistic regression analysis examining factors affecting maternal exposure as assessed by urinary cotinine (exposed vs. unexposed, based on LOQ - Limits of Quantification)

		Odds ratio	95% CI	*P*-value
Multivariable model	**Paternal smoking**	**7.83**	**2.01–30.57**	**0.003**
	**Paternal education academic vs. non-academic**	**0.27**	**0.072–1.02**	**0.053**
	Paternal age	0.99	0.82–1.18	0.89
	Maternal age	0.94	0.76–1.17	0.59
	Monthly Household Income (Net)	2.42	0.55–10.77	0.24

### Evaluation of the association between maternal urine cotinine and birth outcomes

Linear regression analysis examining birthweight (outcome) and its association with maternal urinary cotinine (ng/mL) and other variables was performed (Table 4). In the multivariable model, maternal urinary cotinine as a continuous variable was inversely associated with newborns’ birth weight (beta estimate − 281.39, p-value = 0.048). Gestational age at delivery was also significantly associated with newborns’ birth weight (beta estimate 140.11, p-value = 0.002). The model was adjusted for maternal age, paternal smoking status, maternal pre-pregnancy BMI, newborn sex, and maternal urinary creatinine. We further explored this association and stratified the analysis by the newborns’ sex. No statistically significant association was found for female newborns. By contrast, for male newborns, maternal urinary cotinine was inversely associated with newborns’ birth weight (beta estimate 470.22, p-value = 0.014). Gestational age at delivery was significantly associated with newborns’ birth weight (beta estimate 212.41, p-value = 0.002).

**Table 4:** Linear regression analysis examining newborn exposure as assessed by birthweight, by maternal urinary cotinine (ng/mL)

	*N*, Model *R*^2^		Beta estimate	*P*-value
Multivariable model	44, 0.349			
		**Maternal urinary cotinine (ng/mL)**	**-281.39**	**0.048**
		**Gestational age**	**140.11**	**0.002**
		Paternal smoking	-121.64	0.287
		Maternal age	0.97	0.937
		Maternal pre-pregnancy BMI	10.48	0.425
		Newborn sex	-74.93	0.506
		Maternal urinary creatinine	0.95	0.478
Multivariable model, stratified by newborn sex - **female**	14, 0.175			
		Paternal smoking	-140.77	0.64
		Maternal age	4.25	0.91
		Maternal pre-pregnancy BMI	9.99	0.79
		Gestational age	49.67	0.51
		Maternal urinary cotinine (ng/mL)	25.69	0.94
		Maternal urinary creatinine	-1.17	0.67
	29, 0.529			
Multivariable model, stratified by newborn sex - **male**		**Maternal urinary cotinine (ng/mL)**	**-470.22**	**0.014**
		**Gestational age**	**212.41**	**0.002**
		Paternal smoking	-109.1	0.56
		Maternal age	9.48	0.57
		Maternal pre-pregnancy BMI	6.9	0.66
		Maternal urinary creatinine	2.98	0.09

## Discussion

In our study, 94% of women with a smoking partner and nearly 60% of those with a non-smoking partner were exposed to SHS and had urine cotinine levels above the LOQ. Paternal smoking significantly predicted maternal urinary cotinine levels above the LOQ. This is consistent with previously reported from a recent Israeli “Mother, Child and the Environment” study of pregnant women from the Jerusalem area [11], where paternal smoking was also significantly associated with maternal urinary cotinine (above or below the LOQ) with paternal smoking (*p* < 0.05). A recent population-based study from China found that paternal smoking was independently positively associated with preterm birth [23].

A previous National Israeli survey (RAV MABAT 2015-16) indicated that above 60% of non-smoking adults are exposed to SHS [9]. In our cohort, non-smoking women with smoking partners had significantly higher urinary cotinine levels than those with non-smoking partners, with a total of 77% of non-smoking women having levels of cotinine above the LOQ. In addition to the fact that the overwhelming majority of women with smoking partners had urinary cotinine levels above LOQ (94%), women with smoking partners also had a significantly higher median level of urinary cotinine compared to the “Mother, Child and the Environment” study– 1.13 mg/mL vs. 0.7 mg/mL [11], but lower than in National Survey (RAV MABAT 2015-16)– 1.54 mg/mL [9]. It should be mentioned that in the National Survey (RAV MABAT 2015-16) study the limit of quantification (LOQ) was 1 ng/mL, as in our study the LOQ was 0.5 ng/mL, therefore the differences may be related to this. Moreover, RAV MABAT was conducted on a non-pregnant population. Since it is suggested that cotinine has a faster clearance during pregnancy [24, 25], this difference cannot be explained on the basis of pregnant women versus non-pregnant women. In fact, it is expected that the levels of cotinine in pregnant women would be lower than in non-pregnant women.

Sobh E. et al. also reported in a recent study from Egypt [26] that pregnant women exposed to SHS had a significantly higher urinary cotinine-creatinine ratio than those of non-exposed women. The high prevalence of SHS exposure observed in our cohort exceeds rates reported in many international studies. Reece et al. (2019) found SHS exposure rates during pregnancy ranging from 6 to 73% across 30 countries, with significant regional variations [27]. Specifically in the Middle East and North Africa, the prevalence of daily household SHS exposure is 47.08%, 95%CI (45.17–49.00%). The difference could be attributed to the fact that the study is based on self-reported surveys which are subject to underestimation, as opposed to our study, where the SHS exposure was confirmed by urinary cotinine measures.

In our study, having partners who had completed at least one post-high school degree was significantly associated with maternal urinary cotinine levels below the LOQ. In Japan, a lower level of partner education was associated with indoor smoking, based on data from more than 6,000 partners of non-smoking pregnant women [28]. This finding is consistent with previous reports that lower paternal education is associated with children’s exposure to SHS at home [29–31]. It is known that education level might predict parental nicotine dependence more accurately and some studies have suggested that it is a stronger predictor of SHS exposure than income [29]. This suggests that low paternal education may be a marker for indoor smoking, and may be one of the main barriers to achieving smoke-free homes [29, 32, 33].

The results of these studies show that partners are central to creating and maintaining a smoke-free environment. The approach of developing interventions that work directly with partners and address gender-specific issues underlying fathers’ smoking in the home was reviewed in a recent paper; this differed from the focus of most research, which pertains to mothers’ experiences [34]. A recent systematic review concluded that measures to reduce SHS exposure in nonsmoking pregnant women tend to place the responsibility for “avoidance” on the woman. The authors suggest creating interventions for smoke-free homes or smoking partner cessation [35]. Lately, evidence on the independent role of paternal smoking is accumulating [34], alongside research on the social factors associated with smoking cessation [36, 37]. Recent studies have illuminated important knowledge gaps and cultural contexts. Research from Cairo, Egypt, showed that women in the SHS-exposed group were less likely than other groups to agree that SHS exposure was harmful to their own or their future child’s health [38]. Rodnay and colleagues showed that Israeli pregnant women faced multiple barriers: poor knowledge about SHS risks, misconceptions about effective protective strategies, and lack of support—all contributing to behaviors resulting in high exposure levels [39]. Another Israeli study aimed to identify barriers and facilitators for implementing a smoke-free home and car among expectant fathers [40]. These fathers expressed feeling deeply responsible for their pregnant partners’ health and comfort, insisting they were doing everything possible to minimize their wives’ exposure to secondhand smoke.

Given the high exposure rates observed in our cohort, multilevel interventions are necessary to protect pregnant women from SHS. Healthcare providers should integrate routine screening for SHS exposure into prenatal care protocols. Partner-inclusive approaches that provide smoking cessation support for expectant fathers show modest but promising results [41]. For this reason, campaigns specifically designed for men are needed at the community level to educate them about secondhand smoke dangers, promote quitting smoking, and address gender inequality while empowering women with greater confidence in their ability to avoid secondhand smoke exposure. Working with expectant fathers to establish smoke-free homes and cars seems particularly promising because pregnancy has been identified as a “window of opportunity” that generates a strong motivation and creates a sense of urgency to change smoking behavior while being considered more achievable than smoking cessation. Such targeted approaches recognize the unique role of partners in protecting maternal and fetal health, shifting from individual-focused interventions to those addressing family dynamics and shared responsibility for the unborn child’s wellbeing.

Nevertheless, almost 60% of women with a non-smoking partner had urinary cotinine levels above the LOQ, emphasizing that exposure of pregnant women also comes from sources other than their partner. Therefore, to protect unborn babies, other sources of SHS exposure must be considered. Exposure may have occurred through various potential routes, such as tobacco smoke from neighboring apartments [42], mainly since 74.3% of unexposed women lived in multi-unit buildings, as our study found. The same trend was observed in a recent study, showing that about a third of participating children from non-smoking families were exposed [31]. A survey recently conducted in Israel sought to assess the prevalence of self-reported tobacco smoke incursion (TSI) into private residences [42].

Maternal urinary cotinine levels were inversely associated with newborns’ birth weight. These findings are consistent with the recent finding from the “Mother, Child and the Environment” study, where infants with detectable urinary cotinine had lower birth weight and head circumference [11]. Moreover, in our study, maternal urinary cotinine levels were inversely associated with lower birth weight in male newborns but not in females. Previous studies suggest differences in possible effects of prenatal exposure to SHS between boys and girls, such as anogenital distance [21] or the risk of obesity in early adolescence [22]. The Japan Environment and Children’s Study Group recently examined the relationships between maternal urinary cotinine concentration and placental weight and the ratio of placental to birth weight [43]. A positive nonlinear relationship was observed between maternal cotinine concentration, placental weight, and ratio to birth weight [43]. At higher exposure levels, placental growth response to tobacco smoke differs between male and female offspring - placental weight for males decreased, whereas placental weight flattened out for females. These findings may explain the differences in our cohort between male and female offspring’s birth weight concerning maternal urinary cotinine levels.

In Israel, tobacco control legislation has existed since 1983, with restrictions on smoking in public places and advertising. In December 2018, the Israeli Knesset passed a major amendment to this law, further expanding restrictions on smoking in public areas and regulating advertising, marketing, and packaging of tobacco products [44]. While this legislation represented an important step forward in Israel’s tobacco control efforts [45, 46], it remains insufficient to adequately protect pregnant women and other vulnerable populations from SHS exposure. More comprehensive policy interventions are urgently needed, including legal frameworks to promote and enforce smoke-free zone regulations across all shared residential spaces, such as building entrances, balconies, courtyards, and common areas in apartment buildings.

Our study has several strengths and limitations. Maternal urinary cotinine was measured using a single-spot urine sample. Maternal urine creatinine was measured to correct for variable dilutions among spot samples. The questionnaire collected data on paternal smoking but no other exposures to SHS. The exact location of paternal smoking is unknown (inside/outside). Self-reporting may underestimate true smoking status, leading to misclassification of smoking status [47]. However, a recent study showed that even if smoking was restricted to specific areas (designated areas in the home; balcony; yard; or other), most children in smoking families were measurably exposed to SHS [31]. One of the study’s strengths is that maternal urine samples and questionnaire data were collected simultaneously at birth. This assesses recent exposure to SHS while women were about to deliver. Linear regression was used to examine the association between maternal cotinine levels and birth weight; residual diagnostics supported the use of this parametric model. However, the relatively small sample size may have limited the ability to detect modest associations in some outcomes and multivariable models. Non-significant findings should therefore be interpreted with caution.

## Conclusions

Among participants in our study, 94% of non-smoking pregnant women with a smoking partner had urinary cotinine levels above the LOQ. Maternal urinary cotinine levels at birth are associated with lower birth weight; male newborns might be more affected than females. Our findings support the fact that partners are one of the primary sources of maternal secondhand smoke and may play an essential role in creating and maintaining smoke-free environments.

## Electronic supplementary material

Below is the link to the electronic supplementary material.

Supplementary Material 1

## Data Availability

Data will be available upon reasonable request.

## Abbreviations

aOR: adjusted odd ratio
BMI: body mass index
CI: confidence intervals
EHF: Environmental Health Fund
ETS: Environmental Tobacco Smoke
IQR: interquartile range
LOQ: Limit of Quantification
SD: Standard Deviation
SHS: Second–hand smoke
WHO: World Health Organization
