# Supplementary material for: Paternal smoking and maternal secondhand smoke exposure and the effects on the offspring: results from the EHF (Environmental Health Fund) birth cohort
Source: Isr J Health Policy Res. 2025 Jul 8;14:41. doi: 10.1186/s13584-025-00706-3 (PMC12235883; doi:10.1186/s13584-025-00706-3)
Supplement: Supplementary file 1 — Supplementary Material 1 [file 13584_2025_706_MOESM1_ESM.docx]

### **Additional file 1**

Supplementary Figure 1.

Flow chart of the study population.


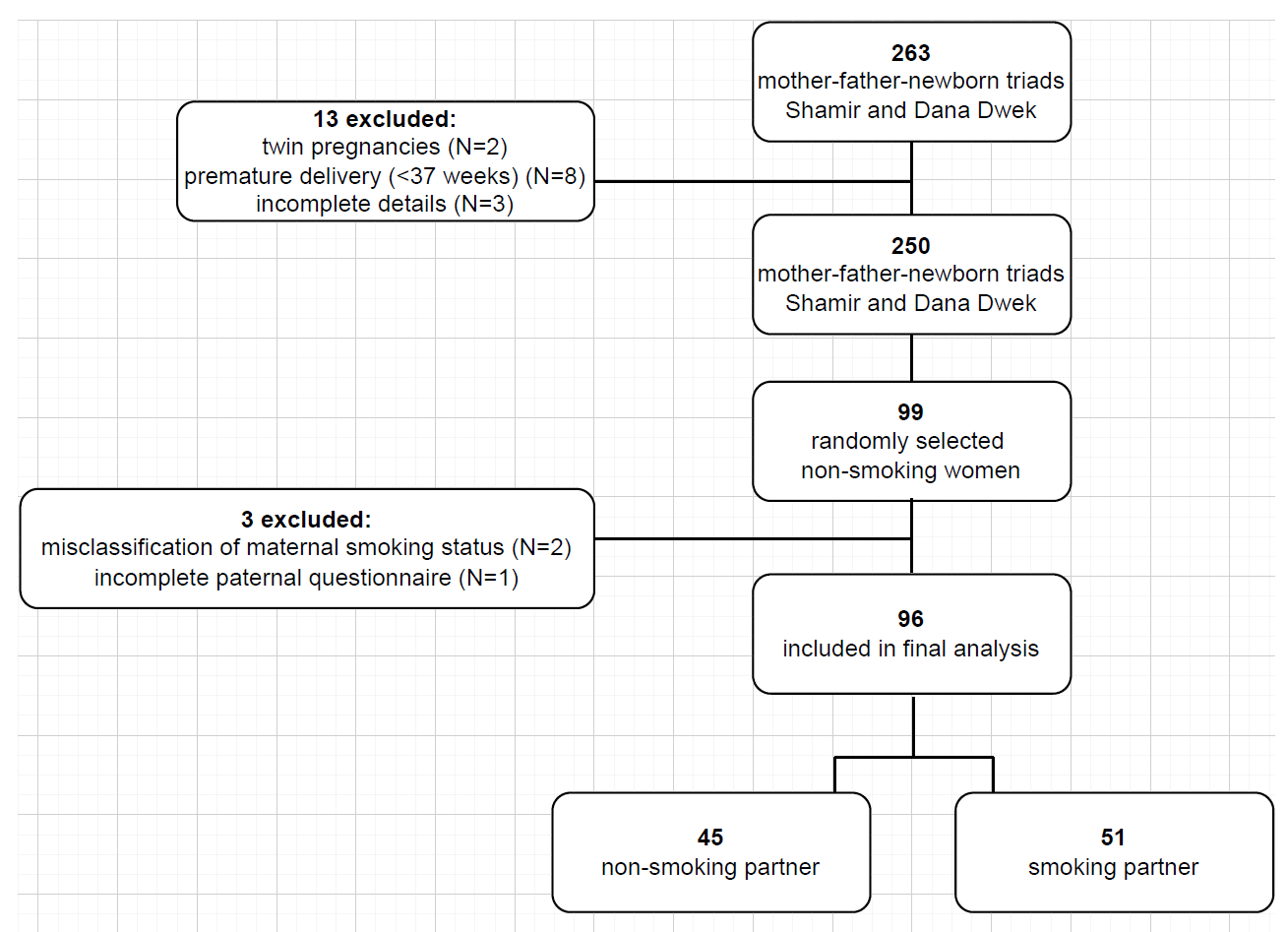


**Supplementary Table 1 - Anthropometric measurements and characteristics of the newborns in the EHF-Assaf-Harofeh-Ichilov birth cohort, Israel, 2013-2015**.

|  | **Self-reported smoking status**  **per questionnaire** | |  | **Urinary Cotinine Below or Above LOQ (0.5 ng/mL)** | |  |
| --- | --- | --- | --- | --- | --- | --- |
| **Maternal characteristics** | **Nonsmoking partner** | **Smoking**  **partner** | **P value** | **Non-exposed** | **Exposed** | **P value** |
| **Number** | **45** | **51** |  | **22** | **74** |  |
| Male (N, %) | 28  62% | 24  47% | 0.137 | 9  40.9% | 43  58.1% | 0.155 |
| Gestational age (weeks)  Mean ± SD | 39.56  1.23 | 39.1  1.28 | 0.081 | 39.25  1.28 | 39.33  1.27 | 0.794 |
| Birth weight (g)  Mean ± SD | 3254  410.8 | 3190  510.9 | 0.509 | 3218  494.2 | 3221  460.4 | 0.984 |
| newborn weight percentile by Dollberg^a^  Mean± SD | 51.68  27.12 | 52.14  28.55 | 0.937 | 52.52  27.66 | 51.75  27.95 | 0.911 |
| Birth length (cm)  Mean ± SD | 49.68  1.74 | 49.39  2.29 | 0.497 | 49.51  2.32 | 49.53  1.97 | 0.964 |
| Head circumference (cm)  Mean ± SD | 34.41  1.18 | 34.23  1.09 | 0.425 | 34.51  1.16 | 34.26  1.13 | 0.369 |
| **Delivery method** |  | | | | | |
| Vaginal (N, %) | 27  64.3% | 26  52% | 0.235 | 12  60% | 41  56.9% | 0.807 |
| C/S (N, %) | 15  35.7% | 24  48% |  | 8  40% | 31  43.1% |  |
| SD - Standard Deviation, LOQ - Limits of quantification;  C/S - cesarean section delivery; SD - Standard Deviation  ^a^ - Dollberg S, Haklai Z, Mimouni FB, Gorfein I, Gordon ES. Birth weight standards in the live-born population in Israel. Isr Med Assoc J. 2005 May;7(5):311-4. | | | | | | |
